# Subtyping *Cryptosporidium ryanae*: A Common Pathogen in Bovine Animals

**DOI:** 10.3390/microorganisms8081107

**Published:** 2020-07-24

**Authors:** Xin Yang, Ni Huang, Wen Jiang, Xinrui Wang, Na Li, Yaqiong Guo, Martin Kváč, Yaoyu Feng, Lihua Xiao

**Affiliations:** 1 Center for Emerging and Zoonotic Diseases, College of Veterinary Medicine, South China Agricultural University, Guangzhou 510642, China; xinyang@webmail.hzau.edu.cn (X.Y.); ni18773655632@163.com (N.H.); pkqdwxr@163.com (X.W.); nli@scau.edu.cn (N.L.); guoyq@scau.edu.cn (Y.G.); 2 Guangdong Laboratory for Lingnan Modern Agriculture, Guangzhou 510642, China; 3 School of Resource and Environmental Engineering, East China University of Science and Technology, Shanghai 200237, China; benjaminxiii@163.com; 4 Institute of Parasitology, Biology Centre of the Academy of Sciences of the Czech Republic, 370 05 České Budějovice, Czech Republic; kvac@paru.cas.cz

**Keywords:** *Cryptosporidium ryanae*, 60-kDa glycoprotein, subtyping tool, host adaptation, geographical differences

## Abstract

*Cryptosporidium ryanae* is one of the most common species for cryptosporidiosis in cattle. However, little is known of the genetic characteristics of *C. ryanae* due to the lack of subtyping tools. In the present study, the 60-kDa glycoprotein (*gp60*) gene of *C. ryanae* was identified in whole genome sequence data and analyzed for sequence characteristics using bioinformatics tools. The protein it encodes had some of the typical characteristics of GP60 proteins, with a signal peptide, a furin cleavage site, and a glycosylphosphatidylinositol anchor at the C terminus of the protein, and numerous O-glycosylation sites. The gene sequence was used in the development of a subtyping tool, which was used in characterizing *C. ryanae* from 110 specimens from dairy cattle, 2 from beef cattle, 6 from yaks, and 4 from water buffaloes in China. Altogether, 17 subtypes from 8 subtype families were recognized, namely XXIa to XXIh. Possible host adaption was identified within this species, reflected by the unique occurrence of XXIa, XXIc, and XXIh in dairy cattle, yaks, and water buffaloes, respectively. Some geographical differences were detected in the distribution of subtype families in dairy cattle; specimens from southern China showed higher genetic diversity than from northern China, and the XXIa subtype family was only seen in dairy cattle in southern and eastern China. The *gp60*-based subtyping tool should be useful in molecular epidemiological studies of the transmission of *C. ryanae*.

## 1. Introduction

*Cryptosporidium* spp. are important pathogens for moderate-to-severe diarrhea in humans and various animals, with a worldwide distribution [1]. Up to date, there are over 40 established species, four of which are commonly seen in cattle, including *Cryptosporidium parvum*, *C. bovis*, *C. ryanae* and *C. andersoni* [2,3]. Among them, *C. parvum* is almost exclusively seen in pre-weaned calves, *C. bovis* and *C. ryanae* mostly in post-weaned calves, and *C. andersoni* in juvenile and adult cattle [4]. These four species differ significantly in infection patterns, pathogenicity, and human infectivity.

Sequence analysis of the 60-kDa glycoprotein (*gp60*) gene has been widely used in subtyping *C. parvum* and other intestinal *Cryptosporidium* species or genotypes. The application of *gp60* subtyping tools has contributed greatly to our understanding of the genetic diversity, transmission dynamics, as well as host adaption in human-pathogenic *Cryptosporidium* spp. [5,6,7,8,9,10,11]. There is also a subtyping tool for *C. andersoni* based on sequence analysis of several genetic loci with simple tandem repeats [12]. Subtyping tools, however, are not available for *C. ryanae* and *C. bovis*, the two bovine-specific intestinal *Cryptosporidium* spp. with reduced pathogenicity.

In the present study, the *gp60* gene of *C. ryanae* was identified in whole-genome sequence data, and used in the development of a subtyping tool for the characterization of isolates from dairy cattle, beef cattle, yaks, and water buffaloes.

## 2. Materials and Methods

### 2.1. Specimens

DNA extracts from 353 *C. ryanae*-positive specimens from dairy cattle, beef cattle, yaks, and water buffaloes on 17 farms in China were used for subtype analysis in the present study (additional files: Table S1). They were from previous and ongoing studies of molecular epidemiology of cryptosporidiosis in bovine animals [13,14,15,16,17,18]. They were confirmed to be positive for *C. ryanae* by PCR and sequence analysis of an ~830-bp fragment of the small subunit (SSU) rRNA gene [19].

### 2.2. Identification of the gp60 Gene of C. ryanae

The whole genome data of one *C. ryanae* isolate (45,019) from a dairy calf in Guangdong, China were used to identify the nucleotide sequence of the *gp60* gene [20]. The *gp60* gene of *C. ryanae* was identified by blastn analysis of the genome assembly with the *gp60* (*cgd6_1080*) gene sequence of *C. parvum*. The coding region of the gene was predicted from the identified contig sequence using FGENESH (http://www.softberry.com/berry.phtml?topic=fgenesh&group=programs&subgroup=gfind). The amino acid sequence predicted was analyzed by a blastp search of the NCBI database to confirm the identification of the *gp60* gene.

### 2.3. Subtyping of C. ryanae

Based on the *gp60* sequence of *C. ryanae*, several sets of primers were designed for nested PCR analysis of the gene (additional files: Table S2). The primer set F1F2 was used in the initial PCR analysis of specimens. Those specimens negative in the PCR analysis were re-analyzed using primer sets F3F4, F3F2, and F5F6. The PCR reaction contained 1 μL of DNA (for the primary PCR) or 2 μL of the primary PCR product (for the secondary PCR), 1× Fermentas Mix (Thermo Scientific, Waltham, MA, USA), and 250 nM primary PCR primers or 500 nM secondary PCR primers in a 50-μL reaction. The amplification was conducted on a T100 PCR thermocycler (Bio-Rad, Hercules, CA, USA) using the following program: A pre-denaturation at 94 °C for 5 min; 35 cycles of 94 °C for 45 s, 55 °C for 45 s, and 68 °C for 1 min; and a final extension at 68 °C for 10 min. The secondary PCR products were visualized under UV after 1.5% (*W*/*V*) agarose gel electrophoresis.

### 2.4. Sequence Analysis

Positive secondary PCR products were sequenced in both directions by Sangon Biotech (Shanghai, China). The sequences generated were assembled, edited, and aligned using ChromasPro V1.5 (http://technelysium.com.au/wp/chromaspro/), BioEdit V7.05 (http://www.mbio.ncsu.edu/bioedit/bioedit), and MUSCLE implemented in MEGA V6.0 (https://www.megasoftware.net/), respectively. Repeat sequences were identified using Tandem Repeat Finder (http://www.tandem.bu.edu/trf/trf). The structure (signal peptide and glycosylphosphatidylinositol (GPI) anchor), N-glycosylated sites, O-glycosylated sites, and furin proteolytic cleavage sites were predicted using the PSORT II (http://psort.hgc.jp/form2.html), NetNGlyc 1.0 (http://www.cbs.dtu.dk/services/NetNGlyc/), YinOYang 1.2 (http://www.cbs.dtu.dk/services/YinOYang/), and ProP 1.0 (http://www.cbs.dtu.dk/services/ProP/) server, respectively. To assess the genetic relationship of *C. ryanae* subtype families, a maximum likelihood tree was constructed using MEGA V6.0 (http://www.megasoftware.net/). The general time-reversible model was used in substitution rate calculations and 1,000 replicates were used in bootstrap analysis. To identify potential recombination among subtype families, DnaSP V5.10 (http://www.ub.es/dnasp/) was used in the calculation of recombination events. Representative nucleotide sequences from the 17 subtypes (four, two, one, one, four, one, three, and one subtype(s) in the XXIa, XXIb, XXIc, XXId, XXIe, XXIf, XXIg, and XXIh subtype families, respectively) obtained in this study were deposited in GenBank under the accession number MT588090-MT588106.

### 2.5. Statistical Analysis

Differences in the distribution of *C. ryanae* XXIa and other subtype families in dairy cattle were analyzed using the χ^2^ test in SPSS V17.0 (IBM, New York, NY, USA). They were considered significant if *p* < 0.01.

## 3. Results

### 3.1. Features of the gp60 Gene of C. ryanae

**Figure 1:**
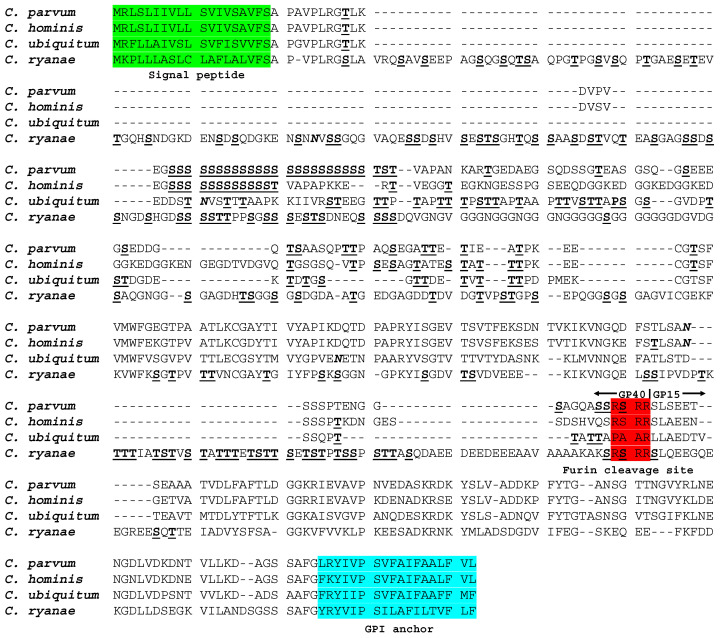
Deduced amino acid sequence of the *gp60* gene of *Cryptosporidium ryanae* (45,019) compared with sequences of *C. parvum* (AF022929), *C. hominis* (FJ839883), and *C. ubiquitum* [10]. Potential N-linked glycosylation sites are indicated in boldface and italic type, and predicted O-linked glycosylation sites are indicated in boldface and underlined type. The first 19 amino acids for the signal peptide and the last 17 amino acids for the GPI anchor are highlighted in green and blue, respectively. The classic furin proteolytic cleavage site sequence RSRR is highlighted in red. Dashes denote amino acid deletions.

The *gp60* gene was identified in contig_13 from the whole-genome sequencing of *C. ryanae* using blastn analysis. Gene prediction using the combination of FGENSH and blastp revealed that the gene was 1548 bp in length and encoded 515 amino acids. It shared significant sequence similarities at the 5′ and 3′ ends with the *gp60* gene of *C. parvum* (AF022929), *C. hominis* (FJ839883), and *C. ubiquitum* [10] at the amino acid level, although the overall sequence similarity was only 19.1–22.4% between *C. ryanae* and the other three species. Despite the much larger size, the structure of the GP60 protein of *C. ryanae* had some of the classic features of GP60 proteins, including the presence of a signal peptide with a cleavage site between Ser 19 and Ala 20, a furin cleavage site (RSRR) between the GP40 and GP15 fragments of the protein, and a glycosylphosphatidylinositol (GPI) anchor at the C terminus of the protein, one potential N-glycosylation site, and 115 O-glycosylation sites. However, unlike *C. parvum* and *C. hominis*, TCA/TCG/TCT trinucleotide repeats were absent in the 5′ region of the *gp60* gene of *C. ryanae*, while GGT trinucleotide repeats encoding a polyglycine tract were recognized by using Tandem Repeat Finder (Figure 1).

### 3.2. Sequence Polymorphisms in the gp60 Gene of C. ryanae

Among the 353 *C. ryanae*-positive specimens in this study, 146 specimens yielded an expected band. For Farm Hezhou, 13 of 37 PCR-positive specimens were randomly selected for sequencing, which generated identical sequences. As a result, PCR products from the remaining 24 specimens were not sequenced. Therefore, 122 *C. ryanae* sequences were used in sequence analysis, including 110 from dairy cattle, 2 from beef cattle, 6 from yaks, and 4 from water buffaloes (Table 1). The nucleotide sequences from 38 specimens were identical to the reference sequence (45,019) from the whole-genome sequencing, while the remaining sequences showed nucleotide differences of 2.1–32.6% (Table 2). The nucleotide differences among divergent sequences were found in several regions with length polymorphism mostly in the form of copy number variations of the trinucleotide repeat. Altogether, 17 subtypes were found among the 122 *gp60* sequences obtained.

### 3.3. Subtype Families and Subtypes of C. ryanae

**Figure 2:**
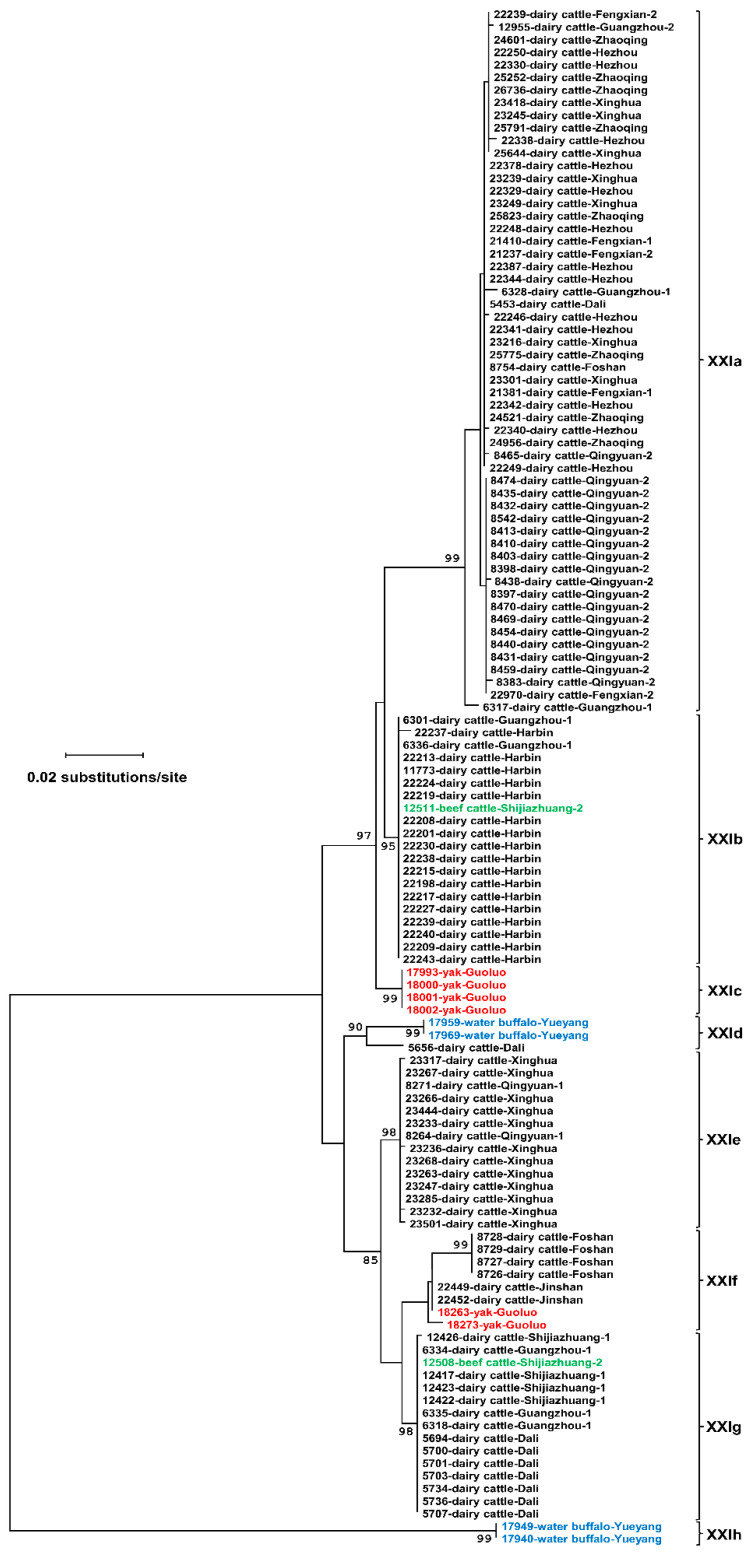
Phylogeny among eight *Cryptosporidium ryanae* subtype families based on a maximum likelihood analysis of the partial *gp60* gene. Sequences from dairy cattle, beef cattle, yaks, and water buffaloes are marked in black, green, red, and blue, respectively. Bootstrap values (>75) are indicated on branches. Scale bar indicates 0.02 nucleotide substitutions per site.

In phylogenetic analysis of the nucleotide sequences generated, the 17 sequence types formed 8 clusters (Figure 2). They were named as the XXIa, XXIb, XXIc, XXId, XXIe, XXIf, XXIg, and XXIh subtype families following the established nomenclature of *gp60* subtype families for *Cryptosporidium* spp. [21]. The *gp60* nucleotide sequences of *C. ryanae* subtype families differed from each other in length by at most 99 bp. Subtype family XXIh formed a highly divergent cluster from the dominant cluster of the remaining seven subtype families (Figure 2). In the dominant cluster, XXIa differed from the remaining six subtype families at both the 5′ and 3′ ends. In contrast, XXIb and XXIc had divergent sequences at the 5′ end, while XXIf and XXIg had divergent sequences at the 3′ end. The sequence differences among subtype families ranged from 2.1% to 32.6% at the nucleotide level (Table 2). DnaSP analysis of the sequences identified six potential recombination events among the eight subtype families (XXIa–XXIh), and five recombination events among the seven more related subtype families (XXIa–XXIg).

**Figure 3:**
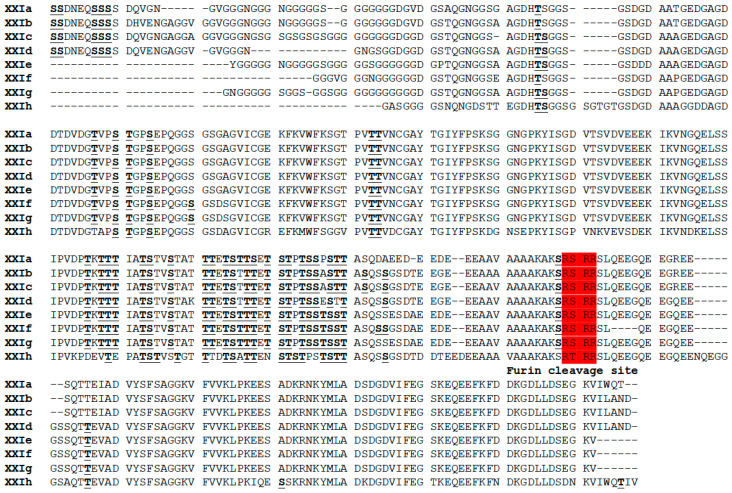
Deduced amino acid sequences of the partial *gp60* gene of eight subtype families (XXIa to XXIh) in *Cryptosporidium ryanae*. Predicted O-linked glycosylation sites are indicated in boldface and underlined type. The classic furin proteolytic cleavage site sequence RSRR/RTRR is shown in red. Dashes denote amino acid deletions.

At the amino acid level, most of the sequence differences among the eight subtype families (XXIa–XXIg) occurred at the N terminus of the partial GP60 sequences (Figure 3). Therefore, all the subtype families had a furin cleavage site. Among the eight subtype families, XXIh had more divergent GP60 sequences, with the furin cleavage site having the motif RTRR instead of the RSRR in other subtype families. In addition, it had five to nine fewer O-glycosylation sites than other subtype families in the GP40 region.

### 3.4. Distribution of C. ryanae Subtypes by Host, Farm, and Geographic Region

Among the eight subtype families of *C. ryanae*, namely XXIa (56), XXIb (20), XXIc (4), XXId (3), XXIe (14), XXIf (8), XXIg (15), and XXIh (2), six were detected in dairy cattle, including XXIa (56), XXIb (19), XXId (1), XXIe (14), XXIf (6), and XXIg (14). XXIa was the predominant subtype family, accounting for about half of the *C. ryanae* specimens from these animals. The distribution of XXIa was significantly different from that of XXIb (χ^2^ = 27.695, *p* = 0.000), XXId (χ^2^ = 71.628, *p* = 0.000), XXIe (χ^2^ = 36.960, *p* = 0.000), XXIf (χ^2^ = 56.145, *p* = 0.000), and XXIg (χ^2^ = 36.960, *p* = 0.000). In contrast, XXIb (1) and XXIg (1), and XXIc (4) and XXIf (2) were detected in small numbers in beef cattle and yaks, respectively. In water buffaloes, only XXId (2) and XXIh (2) were identified in the few PCR-positive specimens (Table 1).

Each farm had one to three subtype families. As shown in Table 1, 10 farms had one subtype family, five farms had two, and two farms had three. On Farm Harbin, all 17 sequences obtained belonged to subtype family XXIb. Similar results were obtained from Farm Hezhou (XXIa) and Qingyuan-2 (XXIa). However, although only two specimens were subtyped on Shijiazhuang-2, they belonged to two subtype families (XXIb and XXIg) (Table 1).

Among the six subtype families (XXIa, XXIb, XXId, XXIe, XXIf, and XXIg) in dairy cattle, five were seen in southern China, including XXIa (44), XXIb (2), XXId (1), XXIe (2), XXIf (4), and XXIg (10). In contrast, three subtype families were found in eastern China, including XXIa (12), XXIe (12), and XXIf (2). However, only two subtype families were detected in northern China, including XXIb (17) and XXIg (4) (Table 3).

## 4. Discussion

Although numerous studies have reported the wide distribution of *C. ryanae* in cattle, little is known about the genetic diversity and transmission of this species with the absence of subtyping tools [4]. In the present study, we identified the *gp60* gene of *C. ryanae* from the recently published whole-genome sequence data [20]. Using the sequence data from the gene, a subtyping tool was established to assess the genetic diversity of *C. ryanae*. The application of this subtyping tool in characterizing *C. ryanae*-positive specimens from dairy cattle, beef cattle, yaks, and water buffaloes has identified possible differences in the distribution of *C. ryanae* subtypes among hosts and geographic areas.

Several major sequence differences are present in the *gp60* gene between *C. ryanae* and other *Cryptosporidium* species. The gene in *C. ryanae* is ~1548 bp in length, much larger than the ~981–1035 bp in *C. parvum* and *C. hominis* [22]. It has low similarity to that of *C. parvum*, *C. hominis*, and *C. ubiquitum* at both the nucleotide and amino acid levels, especially in the *gp40* region. This sequence difference is probably responsible for the failure of PCR to amply the *gp60* locus in *C. ryanae* using the universal primers designed based on the sequences of *C. parvum* and *C. hominis* [21]. Unlike the *gp60* gene of *C. parvum* and other closely related species/genotypes, the TCA/TCG/TCT repeats, which encode a polyserine tract and are used in differentiating subtypes within subtype families, are absent in the *gp60* gene of *C. ryanae*, *C. ubiquitum*, and *C. felis* [5,10,23]. In *C. ryanae*, there is a polyglycine tract encoded by GTT repeats in this region of the *gp60* gene. Otherwise, the GP60 protein of *C. ryanae* has some of the typical features of GP60 proteins of *Cryptosporidium* spp., including a signal peptide at the N terminus, a furin cleavage site between GP40 and GP15, and a glycosylphosphatidylinositol anchor at the C terminus, and over 100 O-glycosylation sites mostly in the GP40 region [22,24].

Based on the *gp60* gene identified, a subtyping tool was developed for *C. ryanae* for the first time. Sequence analysis of 122 PCR-positive specimens led to the identification of 17 subtypes in 8 subtype families. The high genetic diversity is expected, as *C. ryanae* has a high infection rate in cattle and a patent period much longer than *C. parvum* [25,26,27]. This can potentially facilitate the occurrence of genetic recombination among isolates, illustrated in the present study by the identification of genetic recombination events in the overall sequence data and the presence of mosaic sequence patterns among some of the *C. ryanae* subtype families. It is expected that with the use of the newly developed subtyping tool in other areas, other subtype families will be identified. The presence of high sequence heterogeneity in the *gp60* gene of *C. ryanae*, nevertheless, has made PCR amplification of the gene difficult, which together with the large amplicon could be responsible for the poor amplification efficiency of the current subtyping tool.

The subtyping results obtained in the study suggest a possible occurrence of host adaptation within *C. ryanae*. Among the eight subtype families, XXIa is the dominant subtype family in dairy cattle but has thus far not been detected in beef cattle, yaks, and water buffaloes. The numbers of specimens analyzed for the latter, however, are small. Nevertheless, XXIc and XXIh are unique subtype families that have been found only in yaks and water buffaloes in the study, respectively. Previous studies of the SSU rRNA sequences showed the existence of host-adapted *C. ryanae* genotypes in yaks and water buffaloes [16,17,18,28], which is in agreement with the observation of host-adapted subtype families in the present study. In other *Cryptosporidium* spp., host-adapted *gp60* subtype families have been identified in *C. parvum*, *C. hominis*, *C. tyzzeri*, and *C. ubiquitum* [3].

There appears to be some geographic differences in the distribution of *C. ryanae* subtype families in dairy cattle. As shown in Table 3, the genetic diversity of *C. ryanae* in southern China is higher than in northern China, reflected by the presence of three or more subtype families in Guangdong and Yunnan but only one subtype family in Heilongjiang and Hebei. In addition, while XXIa is the most common subtype family in dairy cattle, it is absent from northern China. Instead, XXIb appears to be more common there. In agreement with this, there are also some differences in the prevalence and distribution of *Cryptosporidium* spp. in dairy cattle between northern and southern China. In Heilongjiang, the prevalence of *Cryptosporidium* spp. ranged from 5.3% to 47.7% in dairy cattle, with *C. andersoni* being the predominant species, while in dairy cattle in Guangdong, the prevalence of *Cryptosporidium* spp. ranged from 4.4% to 23.7%, with *C. bovis* as the major species [14,29,30,31,32]. Previously, geographic differences have been reported in the distribution of subtypes of *C. hominis*, *C. parvum*, and *Cryptosporidium* chipmunk genotype I in humans, reflecting differences in the transmission dynamics of those pathogens [8,33].

The genetic diversity in *C. ryanae* could have been underestimated by the current subtyping tool. For some farms, the PCR amplification efficiency at the *gp60* locus was low. For example, on Farm Zhaoqing, only 3 of 66 *C. ryanae* specimens from the second sampling generated the expected products in *gp60* PCR, while on Farm Yangjiang, all 37 *C. ryanae* specimens analyzed were negative at the *gp60* locus. Therefore, there could exist sequence polymorphism in the primer regions in some *C. ryanae* subtypes, which led to the PCR failures [21].

## 5. Conclusions

The *gp60* gene of *C. ryanae* was identified and a subtyping tool was developed based on the nucleotide sequence. The use of the tool in the analysis of fecal specimens revealed high genetic diversity in *C. ryanae*, and the data obtained also provided evidence for the likely occurrence of host adaptation and geographical differences in the distribution of *C. ryanae* subtypes. Further studies are needed to verify these observations and to acquire more whole-genome sequence data from other *C. ryanae* isolates from diverse hosts and geographical areas. This can lead to a comprehensive understanding of the genetic diversity and transmission dynamics of *C. ryanae*.

## Figures and Tables

**Table 1:** *Cryptosporidium ryanae* subtypes identified in bovine animals in China at the *gp60* locus.

Host	Farms	No. of Specimen Analyzed	Subtype Family (No. of Specimen)
Dairy cattle	Harbin	17	XXIb (17)
	Shijiazhuang-1	4	XXIg (4)
	Xinghua	19	XXIa (7), XXIe (12)
	Fengxian-1	2	XXIa (2)
	Fengxian-2	3	XXIa (3)
	Jinshan	2	XXIf (2)
	Dali	9	XXIa (1), XXId (1), XXIg (7)
	Hezhou	13	XXIa (13)
	Guangzhou-1	7	XXIa (2), XXIb (2), XXIg (3)
	Guangzhou-2	1	XXIa (1)
	Qingyuan-1	2	XXIe (2)
	Qingyuan-2	18	XXIa (18)
	Foshan	5	XXIa (1), XXIf (4)
	Zhaoqing	8	XXIa (8)
Beef cattle	Shijiazhuang-2	2	XXIb (1), XXIg (1)
Yaks	Guoluo	6	XXIc (4), XXIf (2)
Water buffalo	Yueyang	4	XXId (2), XXIh (2)
Total		122	XXIa (56), XXIb (20), XXIc (4), XXId (3), XXIe (14), XXIf (8), XXIg (15), XXIh (2)

**Table 2:** Nucleotide sequence similarity among *Cryptosporidium ryanae* subtype families at the *gp60* locus.

	XXIa	XXIb	XXIc	XXId	XXIe	XXIf	XXIg	XXIh
**XXIa**	-							
**XXIb**	95.5	-						
**XXIc**	94.2	97.9	-					
**XXId**	92.0	92.3	91.7	-				
**XXIe**	87.7	87.0	87.1	89.5	-			
**XXIf**	83.6	83.1	83.5	87.1	93.3	-		
**XXIg**	87.0	86.3	86.8	89.6	97.1	94.1	-	
**XXIh**	67.8	67.9	67.4	70.6	71.0	71.8	70.0	-

**Table 3:** Distribution of *Cryptosporidium ryanae* subtype families at the *gp60* locus in dairy cattle by geographical origins.

Geographical Division	Province	No. of Specimen Subtyped	Subtype Family (No. of Specimen)
Northern China	Heilongjiang	17	XXIb (17)
	Hebei	4	XXIg (4)
Eastern China	Jiangsu	19	XXIa (7), XXIe (12)
	Shanghai	7	XXIa (5), XXIf (2)
Southern China	Guangxi	13	XXIa (13)
	Guangdong	41	XXIa (30), XXIb (2), XXIe (2),XXIf (4), XXIg (3)
	Yunnan	9	XXIa (1), XXId (1), XXIg (7)
Total		110	XXIa (56), XXIb (19), XXId (1),XXIe (14), XXIf (6), XXIg (14)

## Supplementary Materials

The following are available online at https://www.mdpi.com/2076-2607/8/8/1107/s1, Table S1: *Cryptosporidium ryanae* specimens used in this study and their *gp60* amplification efficiency; Table S2: Primers for nested PCR analysis of the *gp60* gene in *Cryptosporidium ryanae*.
