# Supplementary material for: Subtyping Cryptosporidium ryanae: A Common Pathogen in Bovine Animals
Source: Microorganisms. 2020 Jul 24;8(8):1107. doi: 10.3390/microorganisms8081107 (PMC7466019; doi:10.3390/microorganisms8081107)
Supplement: Supplementary file 1 [file microorganisms-08-01107-s001.docx]

*Supplementary Material*

Subtyping *Cryptosporidium ryanae*: A Common Pathogen in Bovine Animals

Xin Yang ^1,2^, Ni Huang ^1^, Wen Jiang ^3^, Xinrui Wang ^1^, Na Li ^1^, Yaqiong Guo ^1^, Martin Kváč ^4^, Yaoyu Feng ^1,2,^* and Lihua Xiao ^1,2,^*

^1^ Center for Emerging and Zoonotic Diseases, College of Veterinary Medicine, South China Agricultural University, Guangzhou 510642, China; xinyang@webmail.hzau.edu.cn (X.Y.); ni18773655632@163.com (N.H.); pkqdwxr@163.com (X.W.); nli@scau.edu.cn (N.L.); guoyq@scau.edu.cn (Y.G.)

^2^ Guangdong Laboratory for Lingnan Modern Agriculture, Guangzhou 510642, China

^3^ School of Resource and Environmental Engineering, East China University of Science and Technology, Shanghai 200237, China; benjaminxiii@163.com

^4^ Institute of Parasitology, Biology Centre of the Academy of Sciences of the Czech Republic, České Budějovice 370 05, Czech Republic; kvac@paru.cas.cz

***** Correspondence: yyfeng@scau.edu.cn (Y.F.); lxiao1961@gmail.com (L.X.); Tel.: +86-159-2144-6686 (Y.F.); +86-183-0173-2862 (L.X.)

**Table S1.** *Cryptosporidium ryanae* specimens used in this study and their *gp60* amplification efficiency.

| **Host** | **Farms** | **No. of *C. ryanae*-positive**  **Specimens at SSU rRNA Locus** | **No. of Specimens Positive at *gp60* Locus** | | | | |
| --- | --- | --- | --- | --- | --- | --- | --- |
|  |  |  | **Total** | **Primer**  **F1F2** | **Primer**  **F3F4** | **Primer**  **F3F2** | **Primer**  **F5F6** |
| Dairy cattle | Harbin | 24 | 17 | 9 | 0 | 8 | 0 |
|  | Shijiazhuang-1 | 10 | 4 | 0 | 0 | 3 | 1 |
|  | Xinghua | 19 | 19 | 17 | 1 | 0 | 1 |
|  | Fengxian-1 | 7 | 2 | 2 | 0 | 0 | 0 |
|  | Fengxian-2 | 6 | 3 | 3 | 0 | 0 | 0 |
|  | Jinshan | 2 | 2 | 2 | 0 | 0 | 0 |
|  | Dali | 21 | 9 | 8 | 0 | 1 | 0 |
|  | Hezhou | 46 | 37 | 37 | 0 | 0 | 0 |
|  | Guangzhou-1 | 9 | 7 | 4 | 1 | 2 | 0 |
|  | Guangzhou-2 | 10 | 1 | 0 | 0 | 0 | 1 |
|  | Qingyuan-1 | 9 | 2 | 0 | 0 | 0 | 2 |
|  | Qingyuan-2 | 23 | 18 | 15 | 1 | 1 | 1 |
|  | Foshan | 9 | 5 | 2 | 3 | 0 | 0 |
|  | Yangjiang | 37 | 0 | 0 | 0 | 0 | 0 |
|  | Zhaoqing (1st sampling) | 5 | 5 | 5 | 0 | 0 | 0 |
|  | Zhaoqing (2nd sampling) | 66 | 3 | 3 | 0 | 0 | 0 |
|  | Hainan | 2 | 0 | 0 | 0 | 0 | 0 |
| Beef cattle | Shijiazhuang-2 | 16 | 2 | 1 | 0 | 0 | 1 |
| Yaks | Guoluo | 12 | 6 | 4 | 1 | 0 | 1 |
|  | Yushu | 3 | 0 | 0 | 0 | 0 | 0 |
|  | Haibei | 1 | 0 | 0 | 0 | 0 | 0 |
| Water buffalo | Yueyang | 16 | 4 | 2 | 0 | 0 | 2 |
| Total |  | 353 | 146 | 114 | 7 | 15 | 10 |

**Table S2.** Primers for nested PCR analysis of the *gp60* gene in *Cryptosporidium ryanae*

| **Primer Set** | **Primer** | **Sequence (5′ - 3′)** | **Amplicon Size (bp)** |
| --- | --- | --- | --- |
| F1F2 | Ry-*gp60*-F1 | GCT CGA GTT CTG AGT CGA | 1068 |
|  | Ry-*gp60*-R1 | ATA CCG TTA AAA TGA AGG CCA A |  |
|  | Ry-*gp60*-F2 | CCT CAG ATA ATG AGC AGT CTA | 1024 |
|  | Ry-*gp60*-R2 | GAT GGG ATA ACA TAT CTA TAA CCA AA |  |
| F3F4 | Ry-*gp60*-F3 | TCT ACC GTT CAG ACT GAA GCT | 1165 |
|  | Ry-*gp60*-R1 | ATA CCG TTA AAA TGA AGG CCA A |  |
|  | Ry-*gp60*-F4 | AGT TCT GAT TCA AGT AAC GGT GA | 1106 |
|  | Ry-*gp60*-R2 | GAT GGG ATA ACA TAT CTA TAA CCA AA |  |
| F3F2 | Ry-*gp60*-F3 | TCT ACC GTT CAG ACT GAA GCT | 1165 |
|  | Ry-*gp60*-R1 | ATA CCG TTA AAA TGA AGG CCA A |  |
|  | Ry-*gp60*-F2 | CCT CAG ATA ATG AGC AGT CTA | 1024 |
|  | Ry-*gp60*-R2 | GAT GGG ATA ACA TAT CTA TAA CCA AA |  |
| F5F6 | Ry-*gp60*-F5 | GTC GAC CTC AGG TAA TGA GCA | 1055 |
|  | Ry-*gp60*-R1 | ATA CCG TTA AAA TGA AGG CCA A |  |
|  | Ry-*gp60*-F6 | GTC TAG TTC TTC TGA TCA AGT TG | 1008 |
|  | Ry-*gp60*-R2 | GAT GGG ATA ACA TAT CTA TAA CCA AA |  |
